# Elevated triglycerides and reduced high-density lipoprotein cholesterol are independently associated with the onset of advanced chronic kidney disease: a cohort study of 911,360 individuals from the United Kingdom

**DOI:** 10.1186/s12882-022-02932-2

**Published:** 2022-09-15

**Authors:** Misghina Weldegiorgis, Mark Woodward

**Affiliations:** 1 The George Institute for Global Health, School of Public Health, Imperial College London, London, UK; 2 St George and Sutherland Clinical School, Faculty of Medicine, University of New South Wales Sydney, Sydney, Australia; 3 Department of Epidemiology and Biostatistics, School of Public Health, Imperial College London, London, UK; 4 The George Institute for Global Health, Faculty of Medicine, University of New South Wales Sydney, Sydney, Australia; 5 Department of Epidemiology, Bloomberg School of Public Health, Johns Hopkins University, Baltimore, MD USA

**Keywords:** Dyslipidaemia, Cholesterol, Triglycerides, Estimated glomerular filtration rate (eGFR), Chronic kidney disease

## Abstract

**Background:**

Increased total cholesterol (TC), triglycerides (TG), low-density lipoprotein cholesterol (LDL-C), and decreased high-density lipoprotein cholesterol (HDL-C) concentrations, are established risk factors for cardiovascular morbidity and mortality; but their impact on the risk of advanced chronic kidney disease (CKD) is unclear. This study evaluates the association between the different lipid profiles and the onset of advanced CKD using a general population sample.

**Methods:**

This observational study used records of 911,360 individuals from the English Clinical Practice Research Datalink (from 2000 to 2014), linked to coded hospital discharges and mortality registrations. Cox models were used to examine the independent association between the equal quarters of TC, TG, LDL-C, and HDL-C and the risk of advanced CKD, after adjustment for sex and age, and potential effect mediators.

**Results:**

During a median follow-up of 7.5 years, 11,825 individuals developed CKD stages 4–5. After adjustment for sex and age, the hazard ratios (HRs) and confidence intervals (CIs) for CKD stages 4–5 comparing the 4th vs. 1st quarters of TG and 1st vs. 4th quarters of HDL-C were 2.69 (95% CI, 2.49–2.90) and 2.61 (95% CI, 2.42–2.80), respectively. Additional adjustment for potential effect mediators reduced the HRs to 1.28 (95% CI, 1.15–1.43), and 1.27 (95% CI, 1.14–1.41), respectively. There was no evidence of fully adjusted associations with CKD stages 4–5 for levels of either TC or LDL-C.

**Conclusions:**

Elevated TG and reduced HDL-C levels are independently associated with the onset of advanced CKD. Future studies, such as in basic science and randomized trials, are needed to understand whether associations between TG and HDL-C and the development of CKD are causal.

**Supplementary Information:**

The online version contains supplementary material available at 10.1186/s12882-022-02932-2.

## Background

Chronic kidney disease (CKD) is an emerging major global public health problem, due to its increasing prevalence, poor outcomes, and substantial cost of renal replacement therapy [1]. CKD has multiple risk factors. Some risk factors for CKD, especially hypertension and diabetes, are well established; others are emerging, and yet others are unknown [2]. Thus, identification and treatment of modifiable risk factors remain the best strategy to prevent and delay CKD development.

Dyslipidaemia, including increased total cholesterol (TC), triglycerides (TG), low-density lipoprotein cholesterol (LDL-C), and decreased high-density lipoprotein cholesterol (HDL-C) are risk factors for cardiovascular disease (CVD) [3–9]. Data from several clinical trials have confirmed that treatment with a statin, which lowers LDL-C, substantially reduces the incidence of CVD [10, 11]. However, the association of these different lipids with the development of renal dysfunction remains controversial. Some observational studies have demonstrated a significant association between TC [12], TG [13], LDL-C [12], and HDL-C [14] and the development of CKD, whereas an opposite result was observed in other studies [15, 16]. These inconsistent findings may be attributed to reverse causality, different study populations, different adjustments, different definitions of CKD, and concurrent lipid-lowering therapy.

Given the clinical uncertainty, we sought to determine whether TC, TG, LDL-C, and HDL-C are independently associated with the onset of CKD stages 4–5 using large-scale, prospectively collected data from a general population. Also, we aimed to assess how much of the observed lipid-CKD associations are explained by the established risk factors for CKD.

## Methods

### Study design

The English Clinical Practice Research Datalink (CPRD) provides anonymous data from electronic health records of ≈ 674 primary care practices, covering ≈ 4.4 million active individuals, who represent approximately 8 % of the United Kingdom (UK) population. The CPRD population has been validated to be representative of the UK general population *vis-à-vis* sex, age, and ethnicity [17]. Linkage to area-based social deprivation, Hospitalisation Episode Statistics (HES), and mortality data was available for approximately 75% of all UK CPRD practices. HES database includes admission and discharge dates and diagnostic data (coded with the ICD-10) for several clinical conditions. Scientific approval for our study (15_029R) was given by the Independent Scientific Advisory Committee (ISAC), who govern research using CPRD data.

### Study population

The data used here was formerly used to evaluate the association between body-mass index (BMI) and advanced CKD and end-stage renal disease (ESRD) [18]. It included 1,405,016 individuals from the CPRD database with a valid BMI measurement at any time between January 2000 and March 2014 [18, 19]. Details of the initial inclusion and exclusion criteria is described previously [18, 19]. In brief, eligible individuals had to be aged between 20 to 79 years, had BMI between 15 to 60 kg/m^2^, and had a minimum of 2 years of data prior to their baseline date [19]. Of 1,405,016 individuals, we selected a secondary population of 1,006,629 (71.6%) with baseline data on TC. The first measurement of TC was the index date, and individuals were censored at the earliest date of - death, transfer out of practice, the last collection of quality data for their practice, or the end of the study in March 2014. To minimize reverse causality, we excluded 95,269 (9.5%) who had less than 2 years of follow-up data, leaving 911,360 individuals for analysis.

### Definition of covariates

Covariates were defined at the individual’s baseline or, when not present at that date, the most recent when they were recorded in the previous 2 years. For ease, all are labeled “baseline characteristics”. We characterized smoking status as current or not. The index of multiple deprivation (IMD), a measure of area-based social deprivation, was characterized into equal tenths, where the first implied the least deprived 10%, and the tenth the most deprived 10%, of the English population. We characterised BMI into five groups: < 20, ≥20- < 25, ≥25- < 30, ≥30- < 35, ≥35 kg/m^2^. Comorbidities included prior diabetes mellitus (defined using diabetes diagnosis or treatment-related codes, a prescription of anti-diabetic medication or hemoglobin A_1c_ ≥ 6.5% [≥48 mmol/mol]) and prior CVD (defined using 12 validated cardiovascular outcomes, and includes heart failure, coronary artery, cerebrovascular and peripheral arterial diseases) [18, 20]. Medications included the use of insulin, antihypertensives (renin-angiotensin-aldosterone system inhibitors, thiazides, and any other anti-hypertensive medication), and statin (simvastatin, atorvastatin, and any other statin).

### Definition of predictors and outcomes

Predictors - TC, TG, LDL-C, and HDL-C were determined from laboratory results and were grouped into equal quarters. The outcome of this study was the first record of CKD stages 4–5, derived from internationally recognised clinical definitions and a set of rules integrating mortality records, inpatient procedural or diagnostic codes, and laboratory test/diagnostic results [18, 19]. Where laboratory results were recorded, we calculated estimated glomerular filtration rate (eGFR) from creatinine results using CKD Epidemiology Collaboration (CKD-EPI) formula [21]. CKD stages 4–5 were defined if there were a minimum of two eGFR measurements < 30 mL/min/1.73m^2^, separated by no less than 3 months, with no eGFR result ≥30 mL/min/1.73m^2^ in the intervening period [18, 19].

### Statistical analyses

Baseline characteristics of the secondary population were stratified by the quarters of lipids and presented as means and standard deviations (SDs) for normally distributed variables, but for asymmetric distributions medians and interquartile ranges (25th–75th percentile) were reported. Number and percentage were reported for categorical variables. The associations between the quarters of lipids and the risk of advanced CKD were quantified using complete case Cox proportional hazards analyses. We constructed two models: Model 1 was adjusted for sex and age; Model 2 was adjusted for sex, age, and potential mediators of the effect, comprising smoking status, IMD tenth, systolic blood pressure (SBP), BMI, eGFR, prior diabetes mellitus, prior CVD, antihypertensive medication, insulin, and statin use, at baseline. Pre-specified subgroup analyses by baseline age, sex, BMI, eGFR, diabetes mellitus, CVD, and statin use were performed to evaluate potential effect modification in the adjusted models. The overall and sex-stratified correlations among the different lipids were assessed using Pearson’s correlation coefficients. Furthermore, lipids were analyzed as continuous variables using a restricted spline model adjusted for all the variables in Model 2. The reference values were fixed at the median value of the first quarters - 166, 66.4, and 88.9 mg/dL for TC, TG, and LDL-C, respectively, while for HDL-C, the reference value was fixed at the median value of the fourth quarter, 73.5 mg/dL. All reported *p* values were two-tailed, and *p* values less than 0.05 were considered statistically significant. All statistical analyses used R v3.2.2 (www.R-project.org).

## Results

### Baseline characteristics

Of the 911,360 individuals who had baseline total cholesterol recorded, 53% were women, the mean age of the study population was 54.6 (SD 12.9) years, the mean eGFR was 79.6 (SD 17.7) mL/min/1.73 m^2^ and 12% took statins (Table 1). Baseline characteristics of the individuals, stratified by the quarters of baseline TC, TG, LDL-C, and HDL-C are presented in Table 1 and Supplemental Table S1-S3. Age, SBP, TG, and LDL-C increased with higher TC. The frequency of females and overweight (BMI ≥25 and < 30 kg/m^2^) also increased, whereas eGFR levels and frequencies of prior diabetes mellitus, prior CVD, antihypertensive, insulin, and statin use decreased with higher TC levels (Table 1).

**Table 1:** Baseline characteristics of the main study population across the quarters of baseline total cholesterol

Variables	Total (***n*** = 911,360)	Q1 (***n*** = 227,711)	Q2 (***n*** = 222,006)	Q3 (***n*** = 230,520)	Q4 (***n*** = 231,123)
TC, mg/dL, median [25th–75th %]	213 [182–243]	166 [151–174]	197 [189–205]	224 [217–232]	263 [251–286]
Age, years, mean (SD)	54.6 (12.9)	52.0 (14.6)	53.7 (12.9)	55.5 (12.0)	57.2 (11.3)
Female sex, n (%)	479,498 (52.6)	109,838 (48.2)	113,745 (51.2)	121,932 (52.9)	133,983 (58.0)
Current smoker, n (%)	189,234 (20.8)	47,246 (20.7)	44,875 (20.2)	47,006 (20.4)	50,107 (21.7)
IMD tenth > 5, n (%)	381,892 (41.9)	100,777 (44.3)	92,536 (41.7)	93,717 (40.7)	94,862 (41.0)
BMI, kg/m^2^, mean (SD)	28.5 (5.8)	28.4 (6.2)	28.5 (5.9)	28.7 (5.7)	28.6 (5.3)
BMI category, kg/m^2^, n (%)					
< 20	17,862 (3.2)	6940 (4.5)	4484 (3.2)	3694 (2.7)	2744 (2.1)
≥20, < 25	141,377 (25.0)	42,219 (27.4)	36,159 (25.8)	33,175 (23.8)	29,824 (22.6)
≥25, < 30	214,076 (37.8)	53,723 (34.8)	51,854 (37.0)	54,326 (39.0)	54,173 (41.0)
≥30, < 35	120,029 (21.2)	30,535 (19.8)	29,134 (20.8)	30,366 (21.8)	29,994 (22.7)
≥35	72,311 (12.8)	20,779 (13.5)	18,508 (13.2)	17,686 (12.7)	15,338 (11.6)
SBP, mmHg, mean (SD)	139 (20.9)	134 (19.9)	138 (20.5)	141 (20.7)	144 (21.2)
TG mg/dL, median [25th–75th %]	124 [85.0–177]	97.4 [70.9–136]	112 [79.7–159]	126 [88.6–181]	159 [112–230]
LDL-C, mg/dL, median [25th–75th %]	128 [104–155]	88.9 [77.3–103]	120 [108–128]	143 [131–151]	174 [160–193]
HDL-C, mg/dL, median [25th–75th %]	53.8 [42.9–65.0]	49.5 [40.2–58.0]	53.4 [42.9–64.2]	54.1 [45.6–65.7]	55.7 [46.4–68.1]
eGFR, mL/min/1.73 m^2^, mean (SD)	79.6 (17.7)	82.6 (19.0)	80.7 (17.4)	78.7 (16.8)	76.2 (16.7)
Prior diabetes mellitus, yes, n (%)	93,097 (10.2)	36,985 (16.2)	22,607 (10.2)	18,255 (7.9)	15,250 (6.6)
Prior CVD, yes, n (%)	129,643 (14.2)	49,824 (21.9)	30,272 (13.6)	25,451 (11.0)	24,096 (10.4)
Antihypertensive med., yes, n (%)	317,501 (34.8)	88,945 (39.1)	74,634 (33.6)	76,040 (33.0)	77,882 (33.7)
Insulin, yes, n (%)	19,458 (2.1)	9281 (4.1)	4716 (2.1)	3262 (1.4)	2199 (1.0)
Statin category, n (%)					
Simvastatin	60,696 (6.7)	33,010 (14.5)	13,603 (6.1)	8143 (3.5)	5940 (2.6)
Atorvastatin	34,237 (3.8)	18,448 (8.1)	7690 (3.5)	4327 (1.9)	3772 (1.6)
Other statins	12,345 (1.4)	4462 (2.0)	3249 (1.5)	2562 (1.1)	2072 (0.90

Continuous values shown as mean (standard deviation) or median [interquartile range]; categorical values shown as number (percentage)

*Abbreviations*: *Q* Quarters, *IMD tenth* Index of Multiple Deprivation tenth (First = least deprived, Tenth = most deprived), *BMI* body-mass index, *SBP* systolic blood pressure, *TC* total cholesterol, *TG* triglycerides, *LDL-C* low-density lipoprotein cholesterol, *HDL-C* high-density lipoprotein cholesterol, *eGFR* estimated glomerular filtration rate, *CVD* cardiovascular disease, *med* medication, *Other statins* Cerivastatin, Fluvastatin, Pravastatin, or Rosuvastatin

### Association between quarters of baseline lipids and advanced CKD

During a median follow-up of 7.5 (25th–75th percentile, 4.9–10.1) years, 11,825 individuals developed advanced CKD. The hazard ratios (HRs) and confidence interval (CIs) of the association between the quarters of baseline lipid levels and advanced CKD are shown in Table 2. After accounting for sex and age, the HRs and CIs comparing the 4th vs. 1st quarters of TC, TG, and LDL-C and advanced CKD were 0.62 (95% CI, 0.59–0.65), 2.69 (95% CI, 2.49–2.90), and 0.61 (95% CI, 0.57–0.66), respectively. For HDL-C the HR comparing the 1st vs. 4th quarters and advanced CKD was 2.61 (95% CI 2.42–2.80) (Table 2, Model 1). Adjustment for sex, age, and potential effect mediators reduced all lipid-advanced CKD associations, but the HRs remained significant for TG (HR 4th vs. 1st, 1.28, 95% CI, 1.15–1.43) and HDL-C (HR 1st vs. 4th, 1.27, 95% CI, 1.14–1.41) (Table 2, Model 2).

**Table 2:** Association between the quarters of baseline lipids levels with hazard of CKD stages 4–5

Lipid quarter	No. of events	Total	Hazard ratio (95% confidence interval)	
			Model 1	Model 2
***TC***				
Q1	3489	227,711	**1 (Ref)**	**1 (Ref)**
Q2	2713	222,006	0.74 [0.71–0.78]	1.01
Q3	2673	230,520	0.64 [0.61–0.67]	0.98 [0.90–1.05]
Q4	2950	231,123	0.62 [0.59–0.65]	0.90 [0.83–0.97]
***TG***				
Q1	837	161,250	**1 (Ref)**	**1 (Ref)**
Q2	1552	164,514	1.35 [1.24–1.46]	1.04 [0.93–1.17]
Q3	2325	175,422	1.77 [1.63–1.91]	1.08 [0.97–1.21]
Q4	2953	159,952	2.69 [2.49–2.90]	1.28 [1.15–1.43]
***LDL-C***				
Q1	1607	136,004	**1 (Ref)**	**1 (Ref)**
Q2	9,66	117,132	0.69 [0.63–0.74]	0.92 [0.83–1.03]
Q3	1010	128,509	0.60 [0.55–0.65]	0.91 [0.81–1.03]
Q4	1152	124,492	0.61 [0.57–0.66]	0.95 [0.85–1.07]
***HDL-C***				
Q1	2366	165,744	2.61 [2.42–2.80]	1.27 [1.14–1.41]
Q2	2154	196,406	1.69 [1.57–1.82]	1.10 [1.00–1.22]
Q3	1105	129,916	1.21 [1.11–1.31]	0.99 [0.88–1.11]
Q4	1210	158,641	**1 (Ref)**	**1 (Ref)**

Model 1: adjusted for sex and age; Model 2: adjusted for model 1 plus smoking status, IMD tenth, SBP, BMI, eGFR, prior diabetes mellitus, prior CVD, antihypertensive medication, insulin, and statin use. CKD stages 4–5 were determined if there were at least two eGFR measurements < 30 mL/min/1.73m^2^, separated by at least three months, with no eGFR result ≥30 mL/min/1.73m^2^ in the intervening period

**Fig. 1:**
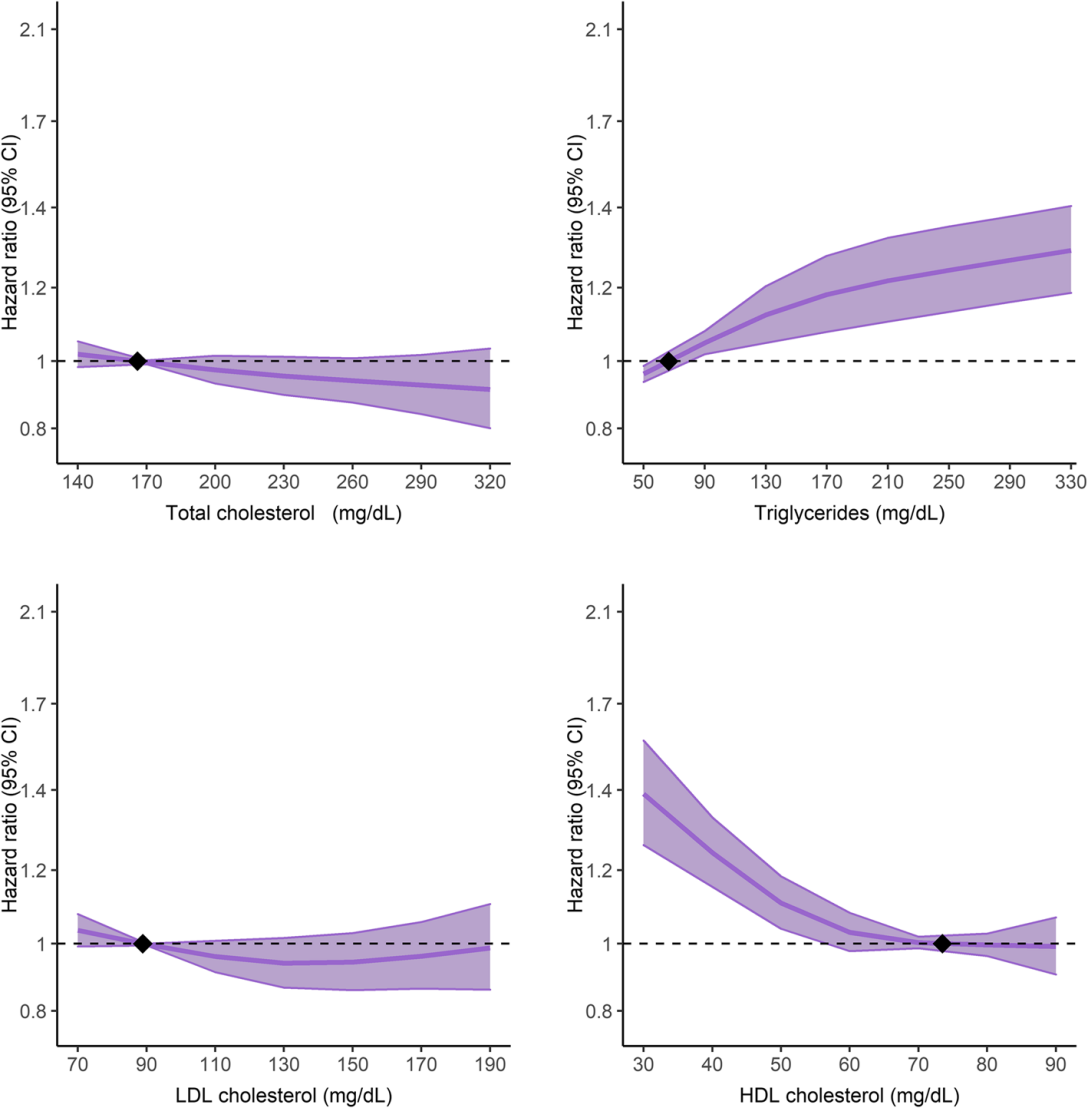
Hazard ratios (95% confidence intervals) for CKD stages 4–5 by baseline lipid levels. The restricted cubic spline of lipids was fitted in a multivariate model adjusting for sex, age, smoking status, IMD tenth, SBP, BMI, eGFR, prior diabetes mellitus, prior CVD, antihypertensive medication, insulin, and statin use

To further evaluate the association between the lipids and advanced CKD, we fitted the lipids as a continuous variable using a restricted spline model adjusting for sex, age, smoking status, IMD tenth, SBP, BMI, eGFR, prior diabetes mellitus, prior CVD, antihypertensive medication, insulin, and statin use. We found an increasing log-linear relationship between TG and advanced CKD, whereas a decreasing association was observed with HDL-C (Fig. 1). In contrast, there were no associations between TC and LDL-C levels and advanced CKD.

**Fig. 2:**
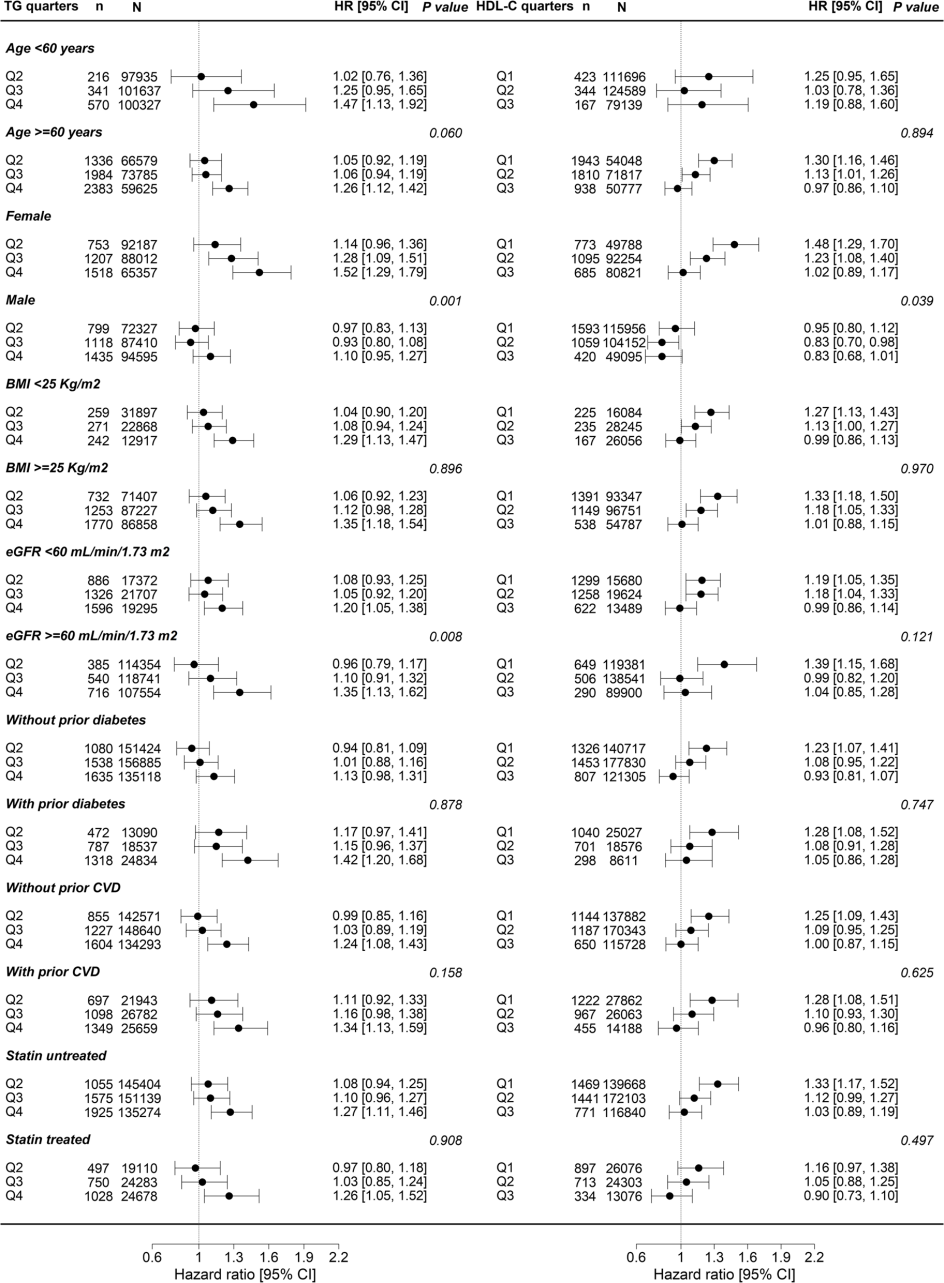
Association between quarters of baseline TG and HDL-C with the hazard of CKD stages 4–5 by pre-defined subgroups of baseline characteristics. The multivariate model was adjusted for sex, age, smoking status, IMD tenth, SBP, BMI, eGFR, prior diabetes mellitus, prior CVD, antihypertensive medication, insulin, and statin use. Q indicates lipid quarters, and the reference value is Q1 and Q4 for TG and HDL-C, respectively. *P* value < 0.05 indicates the presence of significant interaction by baseline sub-groups

The associations between the quarters of both TG and HDL-C with advanced CKD were consistent across subgroups of the baseline characteristics (Fig. 2). Yet, there was evidence of heterogeneity (*p* value for interaction < 0.05) by sex. Visual inspection of the forest plot indicates a stronger association between quarters of TG (4th vs. 1st) and HDL-C (1st vs. 4th) with advanced CKD in females than males. The sex-stratified correlation analyses showed that TG levels are negatively correlated with HDL-C levels in both females (r = − 0.36) and males (r = − 0.35) (Supplemental Table S4).

## Discussion

In this large cohort, nearing a million individuals, with more than 7 years median follow-up we found an independent and graded association between quarters of baseline TG and HDL-C and onset of CKD stages 4–5. The hazard ratio for the highest versus lowest quarters was 1.28 (95% CI, 1.15–1.43) for TG and the lowest versus the highest quarters was 1.27 (95% CI, 1.14–1.41) for HDL-C, after adjustment for sex, age, smoking status, IMD tenth, BMI, SBP, eGFR, prior diabetes mellitus, prior CVD, antihypertensive medication, insulin, and statin use, at baseline. Also, the pattern of the associations between the quarters of TG and HDL-C with CKD stages 4–5 appears to be consistent by pre-defined baseline subgroups, except that there was evidence of a greater effect in women than men. In addition, there was a stronger association between TG and advanced CKD in individuals with eGFR ≥60 mL/min/1.73 m^2^ than eGFR < 60 mL/min/1.73 m^2^. These findings suggest that therapeutic approaches such as fibrates that reduce TG levels and raise HDL-C levels, which were correlated in this study population, may thus reduce the risk of advanced CKD. Conversely, there was no evidence of an association between quarters of TC and LDL-C with risk of CKD stages 4–5, suggesting that lowering TC and LDL-C levels might not have an effect on the prevention of advanced CKD.

Several small studies have found that TG and/or HDL-C levels are associated with the development and/or progression of CKD [13, 22–25]. A systematic review and meta-analysis of longitudinal studies including 30,146 individuals with metabolic syndrome reported that elevated TG and low HDL-C are associated with the development of CKD (eGFR < 60 mL/min per 1.73 m^2^) [22]. Another study found that a higher TG:HDL-C ratio is an independent risk factor for the incidence and progression of CKD [26]. Similarly, a study in 12,549 hypertensive individuals of the China Stroke Primary Prevention Trial (CSPPT) showed that higher TG and TG:HDL-C ratio are independent predictors for rapid renal function decline (eGFR ≥5 mL/min/1.73m^2^ per year) [27]. A study in 12,728 individuals of the Atherosclerosis Risk in Communities (ARIC) cohort showed that high TG and low HDL-C levels are associated with a higher risk of renal dysfunction (serum creatinine ≥0.4 mg/dL) [23]. A study on 15,362 individuals with a baseline eGFR ≥60 mL/min/1.73m^2^ attending Italian diabetes centers, showed that high TG and low HDL-C levels are independent risk factors for the development of diabetic kidney disease [28]. In a community-based, longitudinal cohort study of 2585 individuals, the HDL-C level predicted the development of new-onset kidney disease [24]. In 17,375 individuals of the general Viennese population, lower HDL-C was a predictor of the development of renal dysfunction [25]. In a 2-sample Mendelian randomization study, higher HDL-C was causally related with higher eGFR and lower odds of eGFR < 60 ml/min/1.73 m^2^ per year [14]. A large study in 1,943,682 US veterans with an average eGFR of 74.85 mL/min/1.73 m^2^ showed a U-shaped association between HDL-C levels and renal outcomes [29]. A *post-hoc* analysis of 4326 individuals found that TG levels significantly (*P* value < 0.05) predict the development of proteinuria in both men and women [13]. Further, an analysis of the Action in Diabetes and Vascular Disease: preterAx and diamicroN-MR Controlled Evaluation (ADVANCE) study, a cohort of 11,140 individuals with type 2 diabetes, found that HDL-C is associated with the development of microalbuminuria and macroalbuminuria [30].

Our study, using a large primary care database, confirmed that higher TG and lower HDL-C levels are independently associated with the development of CKD stages 4–5. It is possible that the cholesterol content of triglyceride-rich lipoproteins and low HDL-C levels may lead to inflammation, foam cell formation, atherosclerotic plaques, and ultimately CVD and renal damage [4, 7, 31, 32]. It is also possible that there is a link between low HDL-C, reduced glucose metabolism, and risk of diabetes mellitus [33]. Furthermore, estrogen may have a negative impact on triglycerides contributing to damage to renal structure and function in females [34]. In our study we found a significant interaction by sex, indicating a differential effect of TG and HDL-C in females versus males.

The strengths of our study are the large sample size, long follow-up, well-defined outcome, and subgroup analysis. Additionally, this study compared the magnitude of the risk of CKD stages 4–5 by each component of lipids separately, which might help adopt the best approach for identifying individuals at risk of developing advanced CKD. Furthermore, we omitted the initial 2 years of follow-up from the analysis because underlying diseases might impact negatively on lipid levels, and the observed association between lipid levels and CKD stages 4–5 may be driven by the causal link between the underlying disease stage and a subsequent outcome event. We also acknowledge that this study has limitations. First, a single measurement of each lipid may have resulted in the misclassification of the covariates and the outcome. While non-differential misclassification often results in a bias toward the null, this is not always the case. Second, the completeness of data was another limitation. In our study, 27.5% of individuals had missing TG measurements, 44.5% individuals had missing LDL-C measurements, and 28.6% individuals had missing HDL-C measurements. The decisions to perform cholesterol tests may have been related to confounding by indication if laboratory tests were ordered when individuals presented with illness. Accordingly, individuals with non-measured cholesterol values might differ from those with values recorded. Third, although major risk factors and the issue of reverse causality have been considered, as with any epidemiological study, there are possibilities that residual confounding and selection bias might still exist. Finally, the inclusion of proteinuria would have been helpful as extra confirmation of renal dysfunction, however, it was only available in a small proportion of individuals, so, unfortunately, it was not included in our CKD definition.

## Conclusions

Our study found that elevated TG and reduced HDL-C levels are independently associated with a higher risk of CKD stages 4–5. This suggests that TG and HDL-C levels might be useful for risk stratification as well as for a potential target to reduce the risk of development of CKD. Future studies are needed to understand the potential causal relationship between TG and HDL-C levels and CKD.

## Supplementary Information

**Additional file 1.**

## Data Availability

The anonymized patient-level data analyzed in this study cannot be shared for reasons of information governance. However, data can be accessed by application to Clinical Practice Research Datalink (CPRD), https://www.cprd.com/home/.

## Abbreviations

CKD: Chronic kidney disease
ESRD: End-stage renal disease
TC: Total cholesterol
TG: Triglycerides
LDL-C: low-density lipoprotein cholesterol
HDL-C: High-density lipoprotein cholesterol
IMD: Index of Multiple Deprivation
BMI: Body-mass index
SBP: Systolic blood pressure
eGFR: Estimated glomerular filtration rate
CVD: Cardiovascular disease
CPRD: Clinical Practice Research Datalink
HES: Hospitalisation Episode Statistics
ISAC: Independent Scientific Advisory Committee
HRs: Hazard ratios
CIs: Confidence intervals
SDs: Standard deviations
CSPPT: China Stroke Primary Prevention Trial
ARIC: Atherosclerosis Risk in Communities;
ADVANCE: Action in Diabetes and Vascular Disease: preterAx and diamicroN-MR Controlled Evaluation

## References

[1] GBD 2017 DALYs and HALE Collaborators: Global, regional, and national disability-adjusted life-years (DALYs) for 359 diseases and injuries and healthy life expectancy (HALE) for 195 countries and territories, 1990–2017: A systematic analysis for the global burden of disease study 2017. Lancet 392(10159):1859–1922, 2018. 10.1016/S0140-6736(18)32335-3 PMC6252083 30415748

[2] Levin A, Tonelli M, Bonventre J (2017). Global kidney health 2017 and beyond: A roadmap for closing gaps in care, research, and policy. Lancet.

[3] Lewington S, Whitlock G, Prospective Studies Collaboration (2007). Blood cholesterol and vascular mortality by age, sex, and blood pressure: A meta-analysis of individual data from 61 prospective studies with 55,000 vascular deaths. Lancet.

[4] Nordestgaard BG, Varbo A (2014). Triglycerides and cardiovascular disease. Lancet.

[5] Di Angelantonio E, Sarwar N, Emerging Risk Factors Collaboration (2009). Major lipids, apolipoproteins, and risk of vascular disease. Jama.

[6] Ridker PM (2014). LDL cholesterol: Controversies and future therapeutic directions. Lancet.

[7] Rader DJ, Hovingh GK (2014). HDL and cardiovascular disease. Lancet.

[8] Toth PP, Barter PJ, Rosenson RS (2013). High-density lipoproteins: A consensus statement from the national lipid association. J Clin Lipidol.

[9] Goff DC, Lloyd-Jones DM, Bennett G (2014). 2013 ACC/AHA guideline on the assessment of cardiovascular risk: A report of the american college of cardiology/american heart association task force on practice guidelines. Circulation.

[10] Baigent C, Blackwell L, Cholesterol Treatment Trialists' (CTT) Collaboration (2010). Efficacy and safety of more intensive lowering of LDL cholesterol: A meta-analysis of data from 170,000 participants in 26 randomised trials. Lancet.

[11] Mihaylova B, Emberson J, Cholesterol Treatment Trialists' (CTT) Collaborators (2012). The effects of lowering LDL cholesterol with statin therapy in people at low risk of vascular disease: Meta-analysis of individual data from 27 randomised trials. Lancet.

[12] Schaeffner ES, Kurth T, Curhan GC (2003). Cholesterol and the risk of renal dysfunction in apparently healthy men. J Am Soc Nephrol.

[13] Tozawa M, Iseki K, Iseki C, Oshiro S, Ikemiya Y, Takishita S (2002). Triglyceride, but not total cholesterol or low-density lipoprotein cholesterol levels, predict development of proteinuria. Kidney Int.

[14] Lanktree MB, Theriault S, Walsh M, Pare G (2018). HDL cholesterol, LDL cholesterol, and triglycerides as risk factors for CKD: A mendelian randomization study. Am J Kidney Dis.

[15] Chawla V, Greene T, Beck GJ (2010). Hyperlipidemia and long-term outcomes in nondiabetic chronic kidney disease. Clin J Am Soc Nephrol.

[16] Rahman M, Yang W, Akkina S (2014). Relation of serum lipids and lipoproteins with progression of CKD: The CRIC study. Clin J Am Soc Nephrol.

[17] Herrett E, Gallagher AM, Bhaskaran K (2015). Data resource profile: Clinical practice research datalink (CPRD). Int J Epidemiol.

[18] Herrington WG, Smith M, Bankhead C, et al. Body-mass index and risk of advanced chronic kidney disease: Prospective analyses from a primary care cohort of 1.4 million adults in england. PLoS One 12(3):e0173515. 2017. 10.1371/journal.pone.0173515 PMC5342319 28273171

[19] Weldegiorgis M, Smith M, Herrington WG, Bankhead C, Woodward M (2020). Socioeconomic disadvantage and the risk of advanced chronic kidney disease: Results from a cohort study with 1.4 million participants. Nephrol Dial Transplant.

[20] Rapsomaniki E, Timmis A, George J (2014). Blood pressure and incidence of twelve cardiovascular diseases: Lifetime risks, healthy life-years lost, and age-specific associations in 1.25 million people. Lancet.

[21] Levey AS, Stevens LA, Schmid CH (2009). A new equation to estimate glomerular filtration rate. Ann Intern Med.

[22] Thomas G, Sehgal AR, Kashyap SR, Srinivas TR, Kirwan JP, Navaneethan SD (2011). Metabolic syndrome and kidney disease: A systematic review and meta-analysis. Clin J Am Soc Nephrol.

[23] Muntner P, Coresh J, Smith JC, Eckfeldt J, Klag MJ (2000). Plasma lipids and risk of developing renal dysfunction: The atherosclerosis risk in communities study. Kidney Int.

[24] Fox CS, Larson MG, Leip EP, Culleton B, Wilson PW, Levy D (2004). Predictors of new-onset kidney disease in a community-based population. Jama.

[25] Obermayr RP, Temml C, Knechtelsdorfer M (2008). Predictors of new-onset decline in kidney function in a general middle-european population. Nephrol Dial Transplant.

[26] Tsuruya K, Yoshida H, Nagata M (2015). Impact of the triglycerides to high-density lipoprotein cholesterol ratio on the incidence and progression of CKD: A longitudinal study in a large japanese population. Am J Kidney Dis.

[27] Zhang X, Wang B, Yang J (2019). Serum lipids and risk of rapid renal function decline in treated hypertensive adults with normal renal function. Am J Hypertens.

[28] Russo GT, De Cosmo S, Viazzi F (2016). Plasma triglycerides and HDL-C levels predict the development of diabetic kidney disease in subjects with type 2 diabetes: The AMD annals initiative. Diabetes Care.

[29] Bowe B, Xie Y, Xian H, Balasubramanian S, Al-Aly Z (2016). Low levels of high-density lipoprotein cholesterol increase the risk of incident kidney disease and its progression. Kidney Int.

[30] Morton J, Zoungas S, Li Q (2012). Low HDL cholesterol and the risk of diabetic nephropathy and retinopathy: Results of the ADVANCE study. Diabetes Care.

[31] Varbo A, Benn M, Tybjaerg-Hansen A, Nordestgaard BG (2013). Elevated remnant cholesterol causes both low-grade inflammation and ischemic heart disease, whereas elevated low-density lipoprotein cholesterol causes ischemic heart disease without inflammation. Circulation.

[32] Tabet F, Rye KA (2009). High-density lipoproteins, inflammation and oxidative stress. Clin Sci (Lond).

[33] Drew BG, Duffy SJ, Formosa MF (2009). High-density lipoprotein modulates glucose metabolism in patients with type 2 diabetes mellitus. Circulation.

[34] Shearer GC, Joles JA, Jones H, Walzem RL, Kaysen GA (2000). Estrogen effects on triglyceride metabolism in analbuminemic rats. Kidney Int.

