# Correction: Elevated triglycerides and reduced high-density lipoprotein cholesterol are independently associated with the onset of advanced chronic kidney disease: a cohort study of 911,360 individuals from the United Kingdom

**DOI:** 10.1186/s12882-022-02948-8

**Published:** 2022-09-21

**Authors:** Misghina Weldegiorgis, Mark Woodward

**Affiliations:** 1 The George Institute for Global Health, School of Public Health, Imperial College London, London, UK; 2 St George and Sutherland Clinical School, Faculty of Medicine, University of New South Wales Sydney, Sydney, Australia; 3 Department of Epidemiology and Biostatistics, School of Public Health, Imperial College London, London, UK; 4 The George Institute for Global Health, Faculty of Medicine, University of New South Wales Sydney, Sydney, Australia; 5 Department of Epidemiology, Bloomberg School of Public Health, Johns Hopkins University, Baltimore, MD USA

**Correction: BMC Nephrol 23, 312 (2022)**

**https://doi.org/10.1186/s12882-022-02932-2**

Following publication of the original article [1], the authors informed us that the confidence interval is missing in **Table 2, Column 5, row Q2**. It should be **1.01 [0.95-1.09]**.

The original article has been corrected.
