# Supplementary material for: Elevated triglycerides and reduced high-density lipoprotein cholesterol are independently associated with the onset of advanced chronic kidney disease: a cohort study of 911,360 individuals from the United Kingdom
Source: BMC Nephrol. 2022 Sep 15;23:312. doi: 10.1186/s12882-022-02932-2 (PMC9479392; doi:10.1186/s12882-022-02932-2)
Supplement: Supplementary file 1 — Additional file 1. [file 12882_2022_2932_MOESM1_ESM.docx]

**Supplemental Material**

**Supplemental Table S1.** Baseline characteristics of the main study population across the quarters of baseline triglycerides

**Supplemental Table S2.** Baseline characteristics of the main study population across the quarters of baseline LDL cholesterol

**Supplemental Table S3.** Baseline characteristics of the main study population across the quarters of inverse baseline HDL cholesterol

**Supplemental Table S4.** Correlations among different lipid profiles for the total population and by sex

**Supplemental Table S1.** Baseline characteristics of the main study population across the quarters of baseline triglycerides

| **Variables** | **Total (n=661,138)** | **Q1 (n=161,250)** | **Q2  (n=164,514)** | **Q3 (n=175,422)** | **Q4 (n=159,952)** |
| --- | --- | --- | --- | --- | --- |
| TG mg/dL, median [25^th^-75^th^ %] | 124  [85.0-177] | 66.4  [53.1-75.3] | 99.2  [91.2-109] | 143  [133-159] | 239  [204-301] |
| Age, years, mean (SD) | 54.4 (12.8) | 51.8 (13.4) | 55.1 (13.0) | 55.8 (12.5) | 54.8 (12.0) |
| Female sex, n (%) | 347,584 (52.6) | 102,028 (63.3) | 92,187 (56.0) | 88,012 (50.2) | 65,357 (40.9) |
| Current smoker, n (%) | 135,360 (20.5) | 26,119 (16.2) | 31,005 (18.8) | 37,030 (21.1) | 41,206 (25.8) |
| IMD tenth >5, n (%) | 276,046 (41.8) | 61,166 (37.9) | 66,126 (40.2) | 74,642 (42.5) | 74,112 (46.3) |
| BMI, kg/m^2^, mean (SD) | 28.6 (5.8) | 26.2 (5.2) | 28.2 (5.7) | 29.5 (5.8) | 30.6 (5.6) |
| BMI category, kg/m^2^, n (%) |  |  |  |  |  |
| <20 | 12,693 (3.1) | 6,727 (6.7) | 3,413 (3.3) | 1,822 (1.7) | 731 (0.7) |
| ≥20, <25 | 102,089 (24.6) | 40,373 (40.0) | 28,484 (27.6) | 21,046 (19.1) | 12,186 (12.2) |
| ≥25, <30 | 156,413 (37.8) | 34,952 (34.6) | 39,892 (38.6) | 43,184 (39.2) | 38,385 (38.5) |
| ≥30, <35 | 88,807 (21.4) | 12,677 (12.5) | 19,619 (19.0) | 26,707 (24.3) | 29,804 (29.9) |
| ≥35 | 54,189 (13.1) | 6,295 (6.2) | 11,894 (11.5) | 17,331 (15.7) | 18,669 (18.7) |
| SBP, mmHg, mean (SD) | 139 (20.7) | 134 (20.6) | 139 (20.6) | 141 (20.4) | 143 (20.1) |
| TC, mg/dL, median [25^th^-75^th^ %] | 213  [182-244] | 193  [166-217] | 205  [182-232] | 217  [189-247] | 232  [203-263] |
| LDL-C, mg/dL, median [25^th^-75^th^ %] | 128  [104-155] | 116  [92.8-139] | 129  [104-155] | 136  [112-162] | 137  [108-166] |
| HDL-C, mg/dL, median [25^th^-75^th^ %] | 53.8  [43.3-65.0] | 61.9  [52.2-74.2] | 55.7  [46.4-65.7] | 50.3  [42.5-59.9] | 45.6  [38.7-53.8] |
| eGFR, mL/min/1.73 m^2^, mean (SD) | 79.6 (17.5) | 83.3 (17.3) | 79.2 (17.2) | 77.8 (17.4) | 78.2 (17.7) |
| Prior diabetes mellitus, yes, n (%) | 66,412 (10.0) | 9,951 (6.2) | 13,090 (8.0) | 18,537 (10.6) | 24,834 (15.5) |
| Prior CVD, yes, n (%) | 89,434 (13.5) | 15,050 (9.3) | 21,943 (13.3) | 26,782 (15.3) | 25,659 (16.0) |
| Antihypertensive med., yes, n (%) | 227,319 (34.4) | 39,454 (24.5) | 54,795 (33.3) | 67,325 (38.4) | 65,745 (41.1) |
| Insulin, yes, n (%) | 12,405 (1.9) | 3,531 (2.2) | 2,729 (1.7) | 2,826 (1.6) | 3,319 (2.1) |
| Statin category, n (%) |  |  |  |  |  |
| Simvastatin | 45,620 (6.9) | 7,773 (4.8) | 11,368 (6.9) | 13,730 (7.8) | 12,749 (8.0) |
| Atorvastatin | 26,090 (4.0) | 3,923 (2.4) | 5,671 (3.5) | 7,632 (4.4) | 8,864 (5.5) |
| Other statins | 9,253 (1.4) | 1,196 (0.7) | 2,071 (1.3) | 2,921 (1.7) | 3,065 (1.9) |
| *Abbreviation*: Q, Quarters; IMD tenth, Index of Multiple Deprivation tenth (First= least deprived, Tenth= most deprived); BMI, body-mass index; SBP, systolic blood pressure; TG, triglycerides; TC, total cholesterol; HDL-C, LDL-C, low-density lipoprotein cholesterol; high-density lipoprotein cholesterol; eGFR, estimated glomerular filtration rate; CVD, cardiovascular disease; med, medication; Other statins, Cerivastatin, Fluvastatin, Pravastatin, or Rosuvastatin. *Note*: Continuous variables are shown as mean (standard deviation) or median [interquartile range]; categorical values shown as number (percentage). | | | | | |

**Supplemental Table S2.** Baseline characteristics of the main study population across the quarters of baseline LDL cholesterol

| **Variables** | **Total  (n=506,137)** | **Q1 (n=136,004)** | **Q2 (n=117,132)** | **Q3 (n=128,509)** | **Q4 (n=124,492)** |
| --- | --- | --- | --- | --- | --- |
| LDL-C, mg/dL, median [25^th^-75^th^ %] | 128  [104-155] | 88.9  [77.0-96.7] | 117  [112-124] | 142  [135-147] | 176  [166-193] |
| Age, years, mean (SD) | 54.2 (12.8) | 52.4 (14.2) | 53.1 (12.9) | 54.7 (12.2) | 56.5 (11.4) |
| Female sex, n (%) | 271,280 (53.6) | 73,906 (54.3) | 62,814 (53.6) | 66,897 (52.1) | 67,663 (54.4) |
| Current smoker, n (%) | 102,542 (20.3) | 27,907 (20.5) | 22,896 (19.5) | 25,682 (20.0) | 26,057 (20.9) |
| IMD tenth >5, n (%) | 213,214 (42.1) | 61,066 (44.9) | 49,355 (42.1) | 52,748 (41.0) | 50,045 (40.2) |
| BMI, kg/m^2^, mean (SD) | 28.5 (5.8) | 28.1 (6.2) | 28.5 (6.0) | 28.7 (5.7) | 28.7 (5.4) |
| BMI category, kg/m^2^, n (%) |  |  |  |  |  |
| <20 | 10,310 (3.2) | 4,661 (5.0) | 2,500 (3.3) | 1,912 (2.4) | 1,237 (1.7) |
| ≥20, <25 | 81,994 (25.5) | 26,843 (28.8) | 19,655 (26.1) | 19,007 (23.8) | 16,489 (22.4) |
| ≥25, <30 | 121,227 (37.6) | 31,655 (34.0) | 27,723 (36.8) | 31,201 (39.1) | 30,648 (41.7) |
| ≥30, <35 | 67,232 (20.9) | 18,063 (19.4) | 15,367 (20.4) | 17,328 (21.7) | 16,474 (22.4) |
| ≥35 | 41,265 (12.8) | 11,957 (12.8) | 10,159 (13.5) | 10,415 (13.0) | 8,734 (11.9) |
| SBP, mmHg, mean (SD) | 138 (20.6) | 134 (19.9) | 137 (20.4) | 140 (20.4) | 142 (20.8) |
| TC, mg/dL, median [25^th^-75^th^ %] | 211  [182-240] | 166  [151-182] | 197  [186-209] | 224  [213-236] | 263  [247-282] |
| TG mg/dL, median [25^th^-75^th^ %] | 115  [79.7-168] | 97.4  [70.9-151] | 106  [79.7-159] | 120  [88.6-168] | 141  [104-186] |
| HDL-C, mg/dL, median [25^th^-75^th^ %] | 54.1  [44.1-65.4] | 54.1  [42.5-66.1] | 54.1  [43.3-65.7] | 53.8  [44.1-63.8] | 54.1  [46.0-61.9] |
| eGFR, mL/min/1.73 m^2^, mean (SD) | 80.3 (17.4) | 82.3 (18.7) | 81.5 (17.3) | 79.6 (16.7) | 77.3 (16.3) |
| Prior diabetes mellitus, yes, n (%) | 44,221 (8.7) | 20,229 (14.9) | 9,662 (8.3) | 8,161 (6.4) | 6,169 (5.0) |
| Prior CVD, yes, n (%) | 62,757 (12.4) | 27,190 (20.0) | 13,109 (11.2) | 11,603 (9.0) | 10,855 (8.7) |
| Antihypertensive med., yes, n (%) | 164,640 (32.5) | 52,739 (38.8) | 36,238 (30.9) | 38,461 (29.9) | 37,202 (29.9) |
| Insulin, yes, n (%) | 7,962 (1.6) | 4,430 (3.3) | 1,691 (1.4) | 1,134 (0.9) | 707 (0.6) |
| Statin category, n (%) |  |  |  |  |  |
| Simvastatin | 34,706 (6.9) | 21,046 (15.5) | 6,627 (5.7) | 3,888 (3.0) | 3,145 (2.5) |
| Atorvastatin | 18,812 (3.7) | 11,596 (8.5) | 3,398 (2.9) | 1,953 (1.5) | 1,865 (1.5) |
| Other statins | 6,746 (1.3) | 2,919 (2.2) | 1,683 (1.4) | 1,207 (0.9) | 937 (0.8) |
| *Abbreviation*: Q, Quarters; IMD tenth, Index of Multiple Deprivation tenth (First= least deprived, Tenth= most deprived); BMI, body-mass index; SBP, systolic blood pressure; LDL, low-density lipoprotein cholesterol; TC, total cholesterol; TG, triglycerides; HDL-C, high-density lipoprotein cholesterol; eGFR, estimated glomerular filtration rate; CVD, cardiovascular disease; med, medication; Other statins, Cerivastatin, Fluvastatin, Pravastatin, or Rosuvastatin. *Note*: Continuous variables are shown as mean (standard deviation) or median [interquartile range]; categorical values shown as number (percentage). | | | | | |

**Supplemental Table S3.** Baseline characteristics of the main study population across the quarters of inverse baseline HDL cholesterol

| **Variables** | **Total  (n=650,707)** | **Q1  (n=165,744)** | **Q2  (n=196,406)** | **Q3  (n=129,916)** | **Q4  (n=158,641)** |
| --- | --- | --- | --- | --- | --- |
| HDL-C, mg/dL, median [25^th^-75^th^ %] | 53.8  [42.9-65.0] | 38.7  [34.8-42.5] | 50.3  [46.4-52.2] | 58.8  [58.0-61.9] | 73.5  [69.6-83.1] |
| Age, years, mean (SD) | 54.3 (12.8) | 52.6 (13.0) | 54.0 (12.9) | 54.9 (12.8) | 56.2 (12.4) |
| Female sex, n (%) | 344,233 (52.9) | 49,788 (30.0) | 92,254 (47.0) | 80,821 (62.2) | 121,370 (76.5) |
| Current smoker, n (%) | 134,806 (20.7) | 43,890 (26.5) | 41,242 (21.0) | 23,565 (18.1) | 26,109 (16.5) |
| IMD tenth >5, n (%) | 278,269 (42.8) | 77,962 (47.0) | 86,427 (44.0) | 53,740 (41.4) | 60,140 (37.9) |
| BMI, kg/m^2^, mean (SD) | 28.6 (5.8) | 30.6 (6.0) | 29.2 (5.7) | 27.8 (5.5) | 26.0 (4.9) |
| BMI category, kg/m^2^, n (%) |  |  |  |  |  |
| <20 | 12,955 (3.1) | 1,173 (1.1) | 2,405 (1.9) | 2,845 (3.5) | 6,532 (6.7) |
| ≥20, <25 | 103,193 (25.0) | 14,911 (13.6) | 25,840 (20.7) | 23,211 (28.7) | 39,231 (40.5) |
| ≥25, <30 | 155,660 (37.8) | 40,921 (37.4) | 49,429 (39.5) | 31,453 (38.9) | 33,857 (34.9) |
| ≥30, <35 | 87,369 (21.2) | 30,643 (28.0) | 29,401 (23.5) | 15,178 (18.8) | 12,147 (12.5) |
| ≥35 | 53,074 (12.9) | 21,781 (19.9) | 17,918 (14.3) | 8,156 (10.1) | 5,219 (5.4) |
| SBP, mmHg, mean (SD) | 139 (20.7) | 139 (19.8) | 139 (20.5) | 139 (21.0) | 138 (21.5) |
| TC, mg/dL, median [25^th^-75^th^ %] | 213  [182-244] | 201  [170-232] | 213  [182-244] | 216  [186-247] | 220  [194-251] |
| TG mg/dL, median [25^th^-75^th^ %] | 121  [83.3-177] | 167  [115-239] | 133  [94.8-186] | 106  [79.7-151] | 88.6  [65.5-120] |
| LDL-C, mg/dL, median [25^th^-75^th^ %] | 128  [104-155] | 127  [101-151] | 131  [108-159] | 131  [107-159] | 124  [101-151] |
| eGFR, mL/min/1.73 m^2^, mean (SD) | 80.1 (17.6) | 81.7 (18.2) | 80.0 (17.5) | 79.3 (17.3) | 79.0 (17.1) |
| Prior diabetes mellitus, yes, n (%) | 59,829 (9.2) | 25,027 (15.1) | 18,576 (9.5) | 8,611 (6.6) | 7,615 (4.8) |
| Prior CVD, yes, n (%) | 83,043 (12.8) | 27,862 (16.8) | 26,063 (13.3) | 14,188 (10.9) | 14,930 (9.4) |
| Antihypertensive med., yes, n (%) | 217,034 (33.4) | 60,742 (36.6) | 67,511 (34.4) | 41,737 (32.1) | 47,044 (29.7) |
| Insulin, yes, n (%) | 11,180 (1.7) | 3,692 (2.2) | 3,094 (1.6) | 1,889 (1.5) | 2,505 (1.6) |
| Statin category, n (%) |  |  |  |  |  |
| Simvastatin | 43,760 (6.7) | 14,663 (8.9) | 14,017 (7.1) | 7,516 (5.8) | 7,564 (4.8) |
| Atorvastatin | 24,112 (3.7) | 8,706 (5.3) | 7,586 (3.9) | 4,030 (3.1) | 3,790 (2.4) |
| Other statins | 8,434 (1.3) | 2,707 (1.6) | 2,700 (1.4) | 1,530 (1.2) | 1,497 (0.9) |
| *Abbreviation*: Q, Quarters; IMD tenth, Index of Multiple Deprivation tenth (First= least deprived, Tenth= most deprived); BMI, body-mass index; SBP, systolic blood pressure; TC, total cholesterol; TG, triglycerides; LDL-C, low-density lipoprotein cholesterol; HDL-C, high-density lipoprotein cholesterol eGFR, estimated glomerular filtration rate; CVD, cardiovascular disease; med, medication; Other statins, Cerivastatin, Fluvastatin, Pravastatin, or Rosuvastatin. *Note*: Continuous variables are shown as mean (standard deviation) or median [interquartile range]; categorical values shown as number (percentage). | | | | | |

**Supplemental Table S4.** Correlations among different lipid profiles for the total population and by sex

|  | **Total** | | | |  | **Female** | | | |  | **Male** | | | |
| --- | --- | --- | --- | --- | --- | --- | --- | --- | --- | --- | --- | --- | --- | --- |
|  | **TC** | **TG** | **LDL-C** | **HDL-C** |  | **TC** | **TG** | **LDL-C** | **HDL-C** |  | **TC** | **TG** | **LDL-C** | **HDL-C** |
| **TC** | 1.00 | 0.32 | 0.91 | 0.22 |  | 1.00 | 0.35 | 0.92 | 0.21 |  | 1.00 | 0.32 | 0.91 | 0.19 |
| **TG** | 0.32 | 1.00 | 0.15 | -0.37 |  | 0.35 | 1.00 | 0.22 | -0.36 |  | 0.32 | 1.00 | 0.09 | -0.35 |
| **LDL-C** | 0.91 | 0.15 | 1.00 | -0.03 |  | 0.92 | 0.22 | 1.00 | -0.07 |  | 0.91 | 0.09 | 1.00 | 0.01 |
| **HDL-C** | 0.22 | -0.37 | -0.03 | 1.00 |  | 0.21 | -0.36 | -0.07 | 1.00 |  | 0.19 | -0.35 | 0.01 | 1.00 |
